# Advancements of non-invasive prenatal testing: the role of obstetricians

**DOI:** 10.3389/fmed.2024.1388481

**Published:** 2024-06-13

**Authors:** Nada Eltabbakh, Yalnaz Mohasin, Rafiea Jeddy

**Affiliations:** Royal College of Surgeons in Ireland (Bahrain), Muharraq, Bahrain

**Keywords:** NIPT, obstetricians role, genetic counseling, chromosomal abnormalities, cell-free fetal DNA

## Abstract

Since its debut in 2011, Non-Invasive Prenatal Testing (NIPT) has continually demonstrated its effectiveness in detecting an expanding number of diseases. NIPT offers a less invasive approach to prenatal chromosomal disease screening, providing prospective parents with vital information to better prepare for their potential pregnancy outcomes. NIPT was primarily designed for screening trisomy 13, 18, and 21. However, its scope has since broadened to encompass microdeletions and autosomal dominant monogenic diseases. Conversely, the normalization of NIPT can have unintended consequences. Some patients opt for NIPT without any medical indications, driven by a desire to remain cautious. This over-screening for chromosomal abnormalities can exacerbate pregnancy-related anxiety, as individuals might feel pressured into taking the test unnecessarily. While NIPT can be highly successful when conducted correctly, it is not infallible, and obstetricians play a crucial role in managing patient expectations. This includes providing genetic counseling to individuals with relevant genetic information regarding their personal and family histories. In the context of NIPT, a bioinformatics analysis is performed on a cell-free DNA (cfDNA) sample extracted from the mother’s placenta to determine the fetal fraction (FF). This FF measurement is vital for quality control and ensuring statistical confidence in the test results. Raising awareness among clinicians about the significance of FF enhances patient care and alleviate concerns about the possibility of failed NIPT. This paper aims to explore the ongoing debates and more specifically the significance and pitfalls of NIPT on a psychosocial and ethical scale, all while highlighting the importance of genetic counseling.

## Introduction

In an attempt to ascertain genetic abnormalities in the fetus, invasive and non-invasive approaches to prenatal genetic testing have been introduced during the past 60 years. Historically, prenatal diagnosis evolved from amniotic fluid cytology in the 1960s to chorionic villi sampling in the 1980s. In 1988, Wald et al. have concluded that the levels of human chorionic gonadotropin levels in maternal serum are elevated in Down’s syndrome, which paved the way to the use of maternal biochemical screening tests (1). However, the introduction of nuchal translucency combined with maternal biochemical screening tests in the 1990s marked a pivotal moment in genetic testing (2) to provide a method to screen for individual risk for certain chromosomal abnormalities and guiding the decision to undergo invasive diagnostic testing.

The discovery of cell-free fetal DNA (cfDNA) in maternal plasma in 1997 laid the foundation for a non-invasive approach to prenatal testing. cfDNA is a type of genetic material found circulating freely in the maternal bloodstream. Unlike conventional DNA contained within cells, these fragments, typically contain fewer than 200 DNA base pairs, originate from cells that have died and released their contents into the maternal blood (3). Throughout pregnancy, the mother’s bloodstream becomes a dynamic mixture of cfDNA intertwined with placental DNA. This method utilizes the extraction of maternal blood, allowing the detection of cfDNA, offering a straightforward means to identify potential issues with the fetus, particularly aneuploidies such as trisomy 13, 18, and 21 (4). This method of testing has revolutionized the field of prenatal diagnosis by offering a less invasive approach compared to traditional methods and reducing fetal losses is noteworthy.

During the past decades, prenatal testing has undergone a remarkable evolution. Since its introduction in 2011, over two million NIPTs have been performed, illustrating its growing popularity and rapid commercialization (5). Subsequent advancements in sequencing cfDNA in maternal plasma, have propelled NIPT into mainstream prenatal screening (3, 6). NIPT offers considerable flexibility, with applicability at three distinct time points: before sonography, after sonography, or following the first-trimester test (7). Each option presents unique advantages, albeit with inherent disadvantages. Although NIPT is considered a safer alternative to invasive prenatal testing, it still necessitates confirmatory invasive methods (e.g., Amniocentesis) if a positive test result is obtained, which can elevate the risk of miscarriage (3). One notable concern is the potential routinization of NIPT, which could undermine the reproductive autonomy of women by imposing additional choices that may not align with their societal or moral values (8).

Obstetricians hold a pivotal role in empowering parents by providing information in a clear, accessible manner, free from complex medical terminology. Effective communication not only fosters understanding but also ensures that parents feel comfortable and supported throughout their pregnancy journey. When obstetricians fall short in creating a conducive environment or fail to establish rapport, it can hinder parents’ ability to make informed choices, leading to uncertainty and unease (9). Striking the right balance is crucial; being overly assertive can leave parents feeling pressured into decisions they may not be ready for or comfortable with. Parents should feel free to discuss their preferences and concerns without feeling coerced or judged. By offering comprehensive information, and respectful guidance, obstetricians empower parents to make decisions that align with their values and preferences, ensuring a positive and informed pregnancy experience (10).

While this critical information empowers parents to make informed decisions about their pregnancy, NIPTs also raise ethical concerns, particularly in geographical regions where reproductive choices do not permit termination even in cases of potentially life-threatening fetal outcomes. The aim of this review is to highlight the process of NIPT testing from initial councelling of pregnant patients to interpreting results with the important role of the obstetrician, especially in regions with strict laws in reproductive autonomy.

## Pre-natal testing: the patient selection process and pre-test counceling

The 21st century has called for a rise in worries about genetic testing, this burden becomes clear in ACOG’s 17 publications regarding genetic issues (11). ACOG guidelines for prenatal care for healthy women with uncomplicated visits calls for a visit every 4 weeks for the first 28 weeks of gestation, every 2 weeks until 36 weeks of gestation, and every week until labor (12). Considering that the average gestation period is 40 weeks (about 9 months), that amounts to 15 visits in under a year. Increasing frequency allows to ensure a smooth pregnancy and adequate patient education, which is an important target in reassurance and building a strong rapport between the obstetrician and the pregnant patient (12). While all these visits are important, the first visit allows the obstetrician to screen the patient for any risk factors that might complicate the pregnancy. After obtaining a detailed history and physical examination, all patients undergo baseline blood investigations, urine culture, and serology for vertically transmissible infections (MMR, Syphilis, Chlamydia, etc.). But most importantly, it also includes an ultrasound for all patients to confirm the pregnancy and obtain a more accurate estimated date of delivery (12).

However, certain patients have risk factors associated with fetal genetic abnormalities that would prompt the obstetrician to conduct more testing. These risk factors include, but are not limited to, increased maternal age (>35 years old), consanguinity, family history of known or suspected genetic condition and a previous child with aneuploidy (11, 13). This brings us to mention the importance of pre-test counseling, which involves an inviting discussion between the patient and the obstetrician. The latter needs to mention the reason for testing, the method of testing (bloodwork, imaging, etc.), accuracy, risks, and benefits. This also involves objectively communicating both the advantages and disadvantages of NIPT in a simplified manner that patients can comprehend, which allows patients to make an informed autonomous decision on whether to undergo genetic testing or not, and if yes which one (11). OBs should emphasize that NIPT is a screening tool, not a diagnostic one, and convey that patients’ perceptions of their risk levels may differ from their actual risk level (14). The obstetrician needs to ensure not to scare the patient into doing the tests but also not to reassure the patient too much that they do not want to do it anymore.

Thus, genetic counseling plays a pivotal role in the successful implementation of Non-Invasive Prenatal Testing (NIPT) in prenatal care. Obstetricians (OBs) must navigate the commercial landscape surrounding NIPTs, ensuring they can effectively select and promote NIPT to patients at risk. Despite 60% of women not hearing about NIPT in a study conducted by Petch et al., all participants seemed eager to learn more (15).

## What is the NIPT process and how can we avoid failure of the test?

Chromosomal abnormalities are a significant concern driving the popularity of NIPT, as they contribute to 10 to 20% of stillbirths and approximately 15% of malformations (16, 17). NIPT has proven that it has detection rates as high as 99.2% for trisomies 13, 18, and 21 as well as false positive rates as low as 0.09% (18). It has also demonstrated a sensitivity of 100% and its lowest specificity being 99.95% for trisomy 13 (19). Thus, NIPT serves as a vital screening tool for abnormalities the aforementioned aneuploidies, while also providing valuable diagnostic information for RhD genotyping and fetal gender determination, thus offering a comprehensive insight into the genetic aspects of the pregnancy (14).

The discovery of cell-free fetal DNA in 1997 became the basis of NIPT, revolving around its detection and analysis. This value, detected as early as 5 weeks into the pregnancy, is pregnancy specific as it is cleared from maternal plasma within hours of delivery (20, 21). However, the reliability of NIPT results depends on the fetal fraction, which is the ratio of cell-free fetal DNA to the overall free DNA in maternal plasma. On average, this ratio ranges between 6 and 20%. This method is limited by the instances where it has failed. A multi-center cohort study conducted by Suzumori et al. demonstrated a failure rate of 0.32%, of those, 20% had a low fetal fraction and 16.4% had an altered genomic profile due to maternal malignancy (22). While the failure rates are low, NIPT is not infallible. Although NIPT can be conducted as early as 9–10 weeks into the pregnancy, it is important to note that fetal fraction is affected by gestational age (23). Timing is crucial for accurate results, as performing NIPT too early may yield insufficient placental DNA in maternal blood. The amount of fetal cell-free DNA (cfDNA) is typically lower during the first trimester compared to the second, impacting the accuracy of non-invasive prenatal testing (NIPT) (24). A diminished fetal cfDNA fraction in early pregnancy can lead to an increased risk of false negatives in NIPT results, underscoring its importance in the testing process. Another factor is maternal BMI, as it had a negative correlation with the amount of cfDNA (23). While these factors are clear, a controversial factor was maternal age. While some studies revealed it has a negative correlation with fetal fraction, others have revealed it has no effect (23, 25).

In recent years, NIPTs have also extended beyond to detect rare autosomal aneuploidies (RATs) and copy number variants (CNVs) using deeper sequencing and higher level analyses (14, 26). This includes, but is not limited to, DiGeorge Syndrome, Prader-Willi syndrome and cri-du-chat syndrome (27, 28). When conducted on both high and low-risk groups, NIPT-Plus has shown to have a significantly higher positive rate and positive predictive values (PPV) for trisomy 21 in the former group. However, the positive rates and PPV for other chromosomal abnormalities were not significantly different (29). As such, NIPT-Plus could become a more modern, more specific alternative to NIPT.

## Interpretation of results and post-test counseling

NIPT reports typically categorize results as either “high probability” or “low probability” for the chromosomal test conducted (30). Thus, assisting patients in understanding how to interpret results is crucial. In the case of aneuploidies, trisomy 21 increases fetal fraction and trisomies 13 and 18 decrease it. It is also important to understand that failure rates are higher in the case of multiple gestations, elevating up to 8.7% in dichorionic diamniotic twins (30).

**FIGURE 1:**
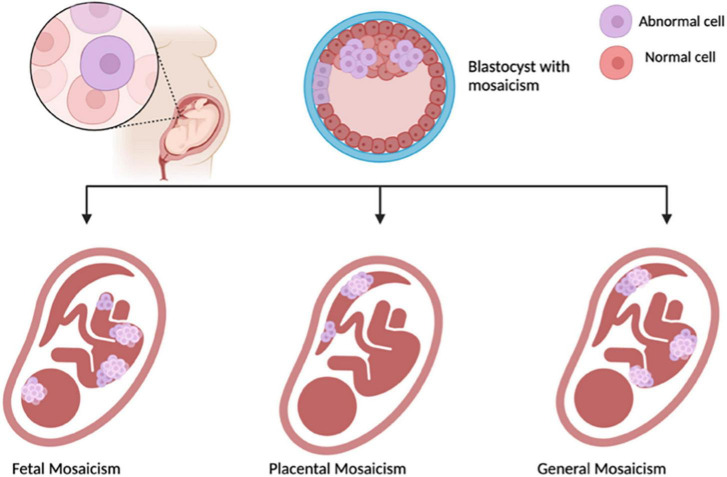
Illustrating Mosaicisim, made with BioRender.com.

Unexplained false-positive results have been reported, and the test’s failure rate is higher in women with multiple gestations, making it unsuitable for this population. Placental mosaicism, a phenomenon where there are differences present between chromosomes of the placenta and the fetus, can add complications. The mosaicism can involve both fetus and placenta, or a combination of the two that can have additional or abnormal chromosomes which can be detected revealing a positive NIPT, when there is no abnormality present (Figure 1). Although generally benign, placental mosaicism can lead to false-positive results, necessitating further testing for accurate diagnoses (31, 32). In these cases, the separation of fetal and placental cells after conception complicates the interpretation of results, highlighting the need for caution in relying solely on NIPT outcomes.

In the 21st century, expectations for quick and conclusive results clash with the reality of NIPT, which could take up to 14 days. And even with the “long” anticipated wait, the test only provides risk estimations and could yield a failed test that would need to be repeated (7). While patients opt to undergo NIPT as a way to obtain a sense of security, waiting for results might induce exhaustion, anxiety and stress. Obstetricians must acknowledge the patients emotional needs. During patient visits, obstetricians are able to provide short-term support, but they also need to look into providing certain patients, especially those with positive NIPT results with longterm care. This can include referrals to mental health counselors, support groups and so on (11). Integration of psychological counseling into antenatal appointments has demonstrated efficacy in mitigating adverse psychological outcomes in many healthcare contexts as well (33).

Patients with a high probability outcome of their NIPT need to undergo further tests that can be invasive such as amniocentesis (34). This calls for further counseling, particularly in this group (35). Furthermore, it is important for the obstetrician to disclose residual risk despite a negative test (11). Even though NIPTs are more widely available in the past decade, there is no protocol for discussion of NIPTs and vary greatly from OBs, thus recommendations are made to create guidelines for discussion and tailoring it further to meet the needs of the patient (36). Consequently, healthcare professionals are caught in a clinically moral dilemma between which test to appoint their pregnant patients all while respecting patient autonomy. As found in interviews conducted by Perrot et al., French healthcare workers have requested implementation of a regulated NIPT program, as well as a unified source of information providing all patients with the same information, as well as further professional training (37).

Genetic counseling plays a critical role in providing comprehensive information, guidance, and support throughout the NIPT process, ensuring informed decision-making and appropriate follow-up measures. There are multiple genetic counseling models that providers can follow: primary counseling provided by the obstetrician, primary counseling provided by the obstetrician in conjunction with supplementary genetic resources, and primary counseling provided by a genetic counselor. The choice of how to proceed is dependent on patient population, provider expertise, infrastructure, and clinic resources (11).

## Discussion

Increasing patient awareness of NIPT is a critical aspect through educational initiatives and should be developed to inform individuals about the benefits, limitations, and implications of NIPT to ensure informed decision-making during pregnancy (26). In a study conducted by Van der Miejj et al., shed light on the complex dynamics that influence a woman’s decisions regarding prenatal screening, particularly regarding NIPT testing. Highly educated women of faith often decline screening due to religious beliefs, resulting in a diminished likelihood of making informed choices. Conversely, non-religious women may opt out simply due to indifference. Socioeconomic factors such as cost can impede freedom of choice, as financial constraints may dictate decisions and limit decision-making during pregnancy. This study was limited by language barriers and sampling biases, highlighting the challenges in extrapolating conclusions, particularly regarding women and their pregnancy, especially due to lower education levels. Addressing these complexities is essential infostering equitable access to prenatal screening and promoting truly informed decision-making among all demographics (8).

The consideration of whether the anxiety associated with NIPT is worthwhile emerges in light of its commercialization. While the test is often promoted to pregnant individuals deemed low risk, some report heightened stress without substantial additional information. Obstetricians must strive to create more welcoming and patient-tailored consultations, avoiding unnecessary tests, and acknowledging the inherent stress of pregnancy. The absence of standardized guidelines for discussing NIPT testing complicates the concept of informed choice, with obstetricians playing a pivotal role in shaping patient decisions. Concerns arise regarding the need for a uniform approach to presenting information, ensuring patients have the necessary knowledge to make decisions autonomously (8). Although there is stress surrounding waiting for results, some individuals find a sense of control through NIPT, while others stated in the study by Stevens et al. that informed consent and giving parents autonomy for decisions regarding their pregnancy is essential to undergo non-invasive testing, but also based on the results determined (36).

When genetic testing occurs during pregnancy, swift communication of results is imperative to allow sufficient time for consideration of reproductive options, including termination (11). While NIPT provides a choice for termination of pregnancy based on fetal abnormalities in many countries, the role of NIPT becomes uncertain in areas where abortion is prohibited due to religious constraints. Additionally, NIPT in countries where abortion laws allow for termination based on gender, contemplation on societal acceptance of individuals with disabilities, which appears to be higher in countries that restrict abortion (38). The challenge of implementing NIPT in rural areas is multifaceted. Basic prenatal care must be prioritized, addressing the lack of support and resources for women in remote regions, particularly those living in poverty with limited access to vaccinations and prenatal supplementation (38). Furthermore, in economically disadvantaged areas, NIPT gives parents the choice to consider their options in the event of a positive diagnosis. Countries such as Ethiopia have grappled with higher rates of childhood mortality, where conditions that are seemingly manageable such as blindness often prove fatal. It is evident that the proliferation of NIPTs in low-income areas, amplify the parents’ prerogative to make decisions regarding termination of pregnancy (9). This trend raises an ethical dilemma regarding the widespread adoption of NIPTs, the likelihood of aborting a fetus with identified abnormalities will surge globally promoting a eugenic approach to pregnancy (39).

In this regard, obstetricians play a pivotal role to ensure parents that NIPT is a screening tool and not diagnostic for detecting abnormalities (39). However, concerns persist that expansion in countries with restrictive abortion laws may drive individuals, particularly in marginalized communities such as low income and minority groups with a positive diagnosis to resort to unsafe abortion practices in countries such as the USA (40). Research has also suggested that many parents use NIPTs as guidance and information for themselves rather than the decisive factor to terminate pregnancies (40). Despite guidance from their obstetrician, it is indicative to emphasize that all women require access to not just support regarding their results, but make informed decisions that take their experience, their circumstances and personal choices following their decision with their NIPT results (41).

Looking forward, advancements in genomic methods, such as next-generation sequencing, show promise in detecting sub-chromosomal aneuploidies. The potential for these methods to become the norm in the next few years underscores the evolving landscape of prenatal diagnostics and raises questions about the future direction of genetic screening technologies. However, literature on the psychological effects of waiting for results remains insufficient. It is also important for future guidelines to exist for obstetricians on how to tailor their discussions with their patients. While we are investigating the advancements of NIPT, it is also important to investigate how patients’ perceptions have evolved with time.

## Author contributions

NE: Writing – original draft, Writing – review and editing. YM: Writing – original draft, Writing – review and editing. RJ: Writing – original draft, Writing – review and editing.
